# How to (not) decide about the motor vs psychomotor origin of psychomotor disturbances in depression

**DOI:** 10.1038/s41380-024-02698-z

**Published:** 2024-10-01

**Authors:** Dusan Hirjak, Stefan Fritze, Sebastian Volkmer, Georg Northoff

**Affiliations:** 1 Department of Psychiatry and Psychotherapy, Central Institute of Mental Health, Medical Faculty Mannheim, University of Heidelberg, Mannheim, Germany; 2 German Centre for Mental Health (DZPG), Partner site Mannheim, Mannheim, Germany; 3 Hector Institute for Artificial Intelligence in Psychiatry, Central Institute of Mental Health, Medical Faculty Mannheim, Heidelberg University, Mannheim, Germany; 4 Mind, Brain Imaging and Neuroethics Research Unit, The Royal’s Institute of Mental Health Research, University of Ottawa, Ottawa, ON Canada

**Keywords:** Neuroscience, Depression

## To the Editor:

Science thrives on hypotheses. We conduct experiments and analyze data to determine whether our hypotheses hold true. Ideally, we work with a set of competing hypotheses where the validity of one negates the others. These hypotheses differ because they produce distinct sets of measures based on their unique underlying assumptions. Concerning the motor hypothesis, one would require local measures limited to the motor cortex like regional homogeneity (ReHo) that measures the local regional synchronization between different voxels within the motor cortex itself independent of any other regions. Another intraregional measure could be the autocorrelation window, which evaluates neural duration and timescale [1, 2]. Under the motor cortex hypothesis, this measure is anticipated to reveal abnormalities, such as prolonged duration, specifically in the motor cortex, but not in other brain regions. Interregional synchronization measured by functional connectivity of the motor cortex with nonmotor regions would be a measure assumed to be abnormal if the psychomotor hypothesis held. Yet another measure in this context would be degree of centrality and global signal correlation that measures the degree to which global brain activity across the whole brain is represented within the local region of the motor cortex. These measures must be analyzed differently due to their differing foundational premises. When applied to the distinction between motor and psychomotor functions, this implies that we need specific measures for each. Neural motor measures are confined to the motor cortex and its intrinsic activity, while neural psychomotor measures extend beyond the motor cortex, including the motor cortex’s activity that depends on neural processes originating elsewhere. The crux of differentiating motor from psychomotor retardation lies not in the mere presence of motor cortex activity, but in distinguishing whether this activity is locally generated within the motor cortex or driven by external neural activity. This distinction between local and global motor cortex activity is fundamental to understanding the motor versus psychomotor models of psychomotor retardation [3, 4].

The very sophisticated study by Wüthrich, Lefebvre et al. [5] seems to confuse that difference and therefore, unjustifiably, doubts and rejects the psychomotor hypothesis. Despite the very large group of major depressive disorder (MDD) patients and state-of-the-art neuroimaging methods, we do believe the results by Wüthrich, Lefebvre et al. [5] are not entirely conclusive because of five important limitations of their research approach, whose consideration in further studies could help to better understand motor and psychomotor abnormalities in mental disorders:

First, the clinical criteria for MDD employed by Wüthrich, Lefebvre et al. [5] need to be mentioned. MDD was defined by a Hamilton Depression Rating Scale (HAMD) score greater than 8, which only indicates mild MDD. The threshold for psychomotor disturbances (PmD) was set even lower: psychomotor retardation (PmR) was identified by a score of ≥1 on HAMD item 8 (“retardation”), psychomotor agitation (PmA) by a score of ≥1 on HAMD item 9 (“agitation”), and concurrent agitation and retardation (PmM) by scores of ≥1 on both items. Furthermore, any healthy controls with a score >1 on these items were excluded, thus eliminating any natural variability in PmD not specific to MDD. It appears the authors compromised the rigor of patient classifications for MDD or PmD to achieve a larger sample size from the cohort, raising questions about the ultimate benefit of this approach.

Second, the selection of solely 18 cortical and subcortical motor regions (e.g. primary motor cortex, supplementary motor area, sensory cortex, superior parietal lobe, caudate, putamen, pallidum, thalamus and cerebellum) is worthy of discussion if the main goal of the study was to investigate the true nature and origin of PmD in MDD. Measuring their local activity is exactly the measure needed to support the motor hypothesis. In contrast, it is not the right kind of measure to refute the psychomotor hypothesis. For that, one would need a measure that relates the selected motor regions’ activity to those regions outside the motor regions themselves, that is, to the non-motor brain regions. Those measures do indeed exist, like the global signal topography with the global signal correlation (GSCORR), that could measure the functional connectivity of the motor regions with the rest of the brain, the non-motor regions [6]. For instance, such analysis of motor cortex GSCORR could, yield no differences between MDD and healthy subjects and could not correlate at all with the degree of the MDD subjects’ PmD. In that case, the psychomotor hypothesis would be refuted in MDD, while, given the local motor cortex findings, the motor hypothesis could be endorsed. Another measure to test the psychomotor hypothesis could consist of testing the raphe nucleus-based functional connectivity to the motor cortex and comparing it to the substantia nigra dopaminergic functional connectivity to the motor cortex [7, 8]. If only the latter but not the former changed in MDD, it would again endorse the motor hypothesis while, at the time, refuting the psychomotor hypothesis. However, the study by Wüthrich, Lefebvre et al. [5] did not test for neither the GSCORR of the motor cortex nor for its raphe nucleus and substantia nigra based functional connectivity related to PmD, nor did they compare any of these measures with their purely local regional approach to the motor system. Therefore, neither their assumption of the motor hypothesis nor their refutation of the alternative psychomotor model of PmD is justified on scientific grounds. Their findings are relevant, showing motor cortex changes being related to PmD, but they do not yet allow to either endorse or refute either of the two hypotheses, e.g., motor and psychomotor. Interpreting their data in this way thus oversteps the boundaries of making proper scientific inferences from the data to their underlying background assumptions, e.g., the models. In conclusion, while it is plausible that alterations in functional connectivity may be addressed with non-invasive brain stimulation targeting areas like M1 or SMA, which are already used in other neuropsychiatric disorders, the results of this study do not support such a conclusion specifically for MDD patients. Further research is needed to validate the efficacy of these interventions in the context of MDD.

Third, antidepressant medications can have varying effects on brain structure and function, and responses to antidepressant medications could vary among MDD patients. Some patients may even experience psychomotor side effects where they may become more retarded, agitated, or restless after starting antidepressant medication. Therefore, researchers should consider the potential heterogeneity in medication response when analyzing their data on MDD patients. We regret the apparent neglect of taking into account medication effects (e.g. antidepressants) by Wüthrich, Lefebvre et al. [5]. One possibility to account for this is by converting individual antidepressant doses to fluoxetine dose equivalents [9].

Fourth, Wüthrich, Lefebvre et al. [5] stated that their *“findings argue against a simple transdiagnostic continuum […] as suggested by Northoff and colleagues”*. However, to be able to answer this question at all, the study cohort would have to be designed for this particular issue and include several diagnostic groups. Such an analysis would have to be corrected for the strength of different symptom continua and would need to analyze the same PmD (e.g. retardation or agitation) across different diagnostic groups as for instance by Magioncalda et al. [8]. Therefore, from our point of view, it is questionable whether they can contradict the transdiagnostic psychomotor model presented by Northoff et al. [3] based on their data.

Fifth, one further significant methodological issue from a clinical perspective warrants critical attention. While we acknowledge that in the context of very large cohorts, an in-depth assessment of PmD may not always be feasible, and researchers might be compelled to rely on individual items from clinical rating scales, using only two items from HAMD is insufficient for distinguishing between motor and psychomotor origins of MDD. Proper differentiation necessitates objective measures and tasks that probe various aspects of pure motor versus psychomotor behavior. This lack of comprehensive assessment compromises the validity and depth of the findings related to PmD in this study. Nevertheless, the authors did not discuss this point in the limitation section of their paper. It would be more interesting and important for further replication studies if the authors took a stand on this issue. Using a single item from the HAMD to examine PmD has four major limitations: (i) PmDs are multifaceted conditions with a wide range of symptoms, including affective, cognitive, and motor disturbances. Using item #8 RETARDATION (slowness of thought and speech, impaired ability to concentrate, decreased motor activity) or item #9 AGITATION of the HAMD might oversimplify the complex nature of PmD in MDD. (ii) A single-item assessment may not provide enough information to make a comprehensive diagnosis of PmD. Reducing this phenomenon to single items #8 and #9 may not fully capture the nuances of the patient’s experience. A full diagnostic evaluation typically requires a broader assessment of multiple PmD-associated symptoms, their severity, and their duration. (iii) Clinicians may differ in their interpretation of a single-item score, leading to potential inconsistencies in assessment. (iv) Focusing on a single item may lead to the oversight of PmD-related symptoms that could inform treatment decisions or be critical for understanding the treatment outcome.

Finally, we think that the results reported by Wüthrich, Lefebvre et al. [5] do not necessarily have to contradict the model of Northoff et al. [3], because both their results and those of Northoff et al. [3] can be noisy in terms of study sample characteristics and methods. We converge to the correct result over a period of time and not by studying a single cohort (which as mentioned by the authors themselves was not designed to measure PmD). To uncover or replicate robust multivariate brain-wide PmD associations, large sample sizes are typically required [10]. However, this need for large sample sizes could impede innovation by prolonging the discovery of new neurobiological mechanisms [11]. It could be contended that data derived from meticulously designed studies with rigorously classified participants [12] might obviate the need for such large cohorts, even in neuroimaging analyses—a viewpoint consistent with the findings of other scholars in the field [12–15].
